# Areolar Carcinoma of Omentum, Simulating Ovarian Tumor

**Published:** 1872-07-01

**Authors:** N. Senn

**Affiliations:** Ashford, Wis.


### AREOLAR CARCINOMA OF OMENTUM, SIMULATING OVARIAN TUMOR.

BY N. SENN, M. D., ASHFORD, WIS.

*Read before the Fond du Lac Co. Medical Society, Dec.* 25, 1871, *and published by request.*

We often obtain great benefit from the mistakes of others.

This rule is applicable not only to merchants, clergymen and lawers, but more than equally so to the medical profession. What an everlasting impression is made upon the mind of the novice in medicine, when he reads in every standard surgical work, the story that no less a man than John Hunter tapped the bladder for ascites, and in Sir Astley Cooper's Lectures he hears of the famous "dry tap." These are lessons never to be forgotten; they have stamped themselves indelibly upon memory's tablets, and present themselves fresh and anew every time he is called upon to treat an obscure abdominal enlargement. If medical men would be more honest, and as anxious to report their mistakes as their correct diagnoses—their unsuccessful cases as well as their successful ones—our field for progress would be a wider and more fertile one, our statistics more extensive and reliable.

Obscure abdominal tumors and enlargements are some of the most difficult and perplexing affections to diagnose. Of this we had lately an instructive illustration in the case presented by myself to the members of this society for examination and advice at the last meeting. I refer to the lady with the abdominal enlargement. As the opinions of those present differed widely as to the nature of her case at the time, it must be interesting for you to hear further from the case.

The patient has since died. A post-mortem examination—which was insisted upon and finally consented to by her relatives and friends—has revealed the true nature of that enlargement. I will, however, attempt first to give a full history of her case.

Mrs. E.; æt. 41 years; no hereditary diseases existing among her family relations. She was the mother of six children; a robust, healthy, active woman. After her second confinement, fourteen years ago, her abdomen remained larger than usual; she suffered some from backache, general debility, and menorrhagia. She obtained no benefit from medical treatment. Two years after, she was delivered of her last child, being now twelve years of age.

The same train of symptoms re-appeared. Menstruation ceased about a year ago. She remained about in the same condition until the latter part of last summer, being able up to this time to attend to her domestic cares, and even work on the farm; from this time, however, she thought her abdomen began to increase in size.

When I first examined her, about two months ago, she appeared like a woman in good health, ruddy complexion and well nourished; pulse 75; respiration normal. She complained of some pain and a feeling of distress in the umbilical region; appetite poor; bowels constipated; no prominent pelvic symptoms.

A physical examination of the abdomen and pelvis presented the following facts: Abdomen nearly as large as at full term of pregnancy; in the erect position it was prominent, projecting forward; dullness on percussion from pubis to umbilicus. In the recumbent position on her back, with the legs drawn up, considerable of the rotundity of the abdomen remained; right iliac region more prominent than the left; percussion dullness more marked and diffuse on right than on the left side, and extending from pubis to near umbilicus; some changes in percussion sound in different positions; the right iliac region, however, remaining perfectly flat. Although no distinct and circumscribed tumor could be satisfactorily felt, still there existed a fullness and sense of resistance from the umbilical to the right iliac region. Fluctuation distinct; uterus slightly prolapsed and pressed forward towards the pubis; uterine sound passes to the depth of three inches; body of uterus easily movable in all directions. Liver, heart and lungs apparently healthy in function and structure; urine scanty, loaded with amorphous urates, containing no bile nor albumen.

With this history of the case, and these physical signs and symptoms, it was difficult to describe whether we had to deal with a simple serous effusion into the peritoneal cavity, or with a large multilocular ovarian cyst. The patient was unable to state when the enlargement first commenced.

On the one hand, the healthy condition of the central organs, and the absence of cancerous tumor or tubercular deposits in any other organ or part of the body, seemed to be against the supposition of ascites; on the other hand, the patient claimed to have suffered from enlargement of the abdomen and pelvis symptoms years before. Various remedies were administered, but all failed to reduce the swelling or even afford temporary relief. The abdomen gradually but steadily increased in size; the general health began to fail.

Soon after you saw her, she was confined to her bed; the pain became excruciating; total loss of appetite; hectic fever and rapid emaciation followed.

On the eighth day of November the abdomen was so much distended, that it materially interfered with respiration. She was now tapped in the ordinary manner and about four gallons of serum removed. The fluid was strongly alkaline, with a specific gravity of 1012°, and on the application of heat and nitric acid deposited half of its volume albumen.

The relief afforded by tapping was only partial; the pains continued in their severity, and required large doses of morphine to moderate them. Soon after the operation the right leg became œdematous; her abdomen refilled rapidly; prostration with profuse sweating followed.

On the 20th of November I was sent for in great haste to tap her again, but before I arrived she had a severe rigor, followed by intense abdominal pains, delirium and collapse, from which she never rallied. She died in the evening of the same day.

POST-MORTEM EXAMINATION TWENTY-FOUR HOURS AFTER DEATH.

Nearly as much fluid was removed as during the first tapping. The serum was of a straw color, and contained patches of lymph.

On opening the abdominal cavity, the parietal peritoneum was found congested, its surface perfectly smooth. The omentum almost over the entire surface presented one diseased mass. It appeared to be infiltrated and covered by a heterologous formation of variable color and consistency. At some points there were masses of a dull white color; at others resembling white jelly; and still others of an almost brownish hue as though blood had been intimately mixed with it. The consistency varied from a jelly like amorphous mass, to the natural density of the tissues in that locality. The omental fat was interspersed in various proportions with these deposits; in some localities it preserved its normal quantity and appearance, while in others it was entirely replaced by this new material. The vascularity was great in some portions, while others were almost devoid of vessels. The omentum was adherent to some of the intestines beneath.

The greater part of the tumor was located in the right groin, where it was so extensive as to press upon and partially obliterate the iliac vein, giving rise to œdema of the right leg during life. The mesenteric glands were only moderately enlarged. All the rest of the abdominal organs, with perhaps increased vascularity, were healthy.

With the microscope, large, round, oval and irregular cells, with large nuclei were found, also free nuclei, and fat globules.

Our literature on this subject appears to be very deficient. I have searched through the contents of several volumes of different medical journals, and have been unable to find a similar case. Some authors fail even to mention it in their text books.

Flint in his "Practice of Medicine," page 454, says, "Cancer of the peritoneum or omentum may occur primarily or by extension. Patients suffering from it may suffer but little pain, and be entirely free from tenderness. It generally proves fatal from one month to a year or more."

Niemeyer in his excellent work, vol. I, page 703, remarks: "Of the different kinds of cancer of the peritoneum the areolar is the only one which is accompanied by ascites. Schirrhus and medullary appear in the form of diffuse granulations over the whole peritoneal surface about the size of a pea. Areolar cancer, on the other hand, sometimes forms colossal tumors, which are almost exclusively formed in and on the omentum.

"The first symptom is the effusion. The diagnosis from ovarian dropsy he mentions to be in some cases extremely difficult and perplexing; even Bamberger's test, the percussion sound in the lumbar region, between the crest of the ilium and the last rib, frequently fails."

Bardeleben in his recent edition of his Extensive System of Surgery, vol. I, page 527, gives a very accurate anatomical and microscopical description of this affection.

"Areolar cancer of the peritoneum or omentum, generally appears as a diffuse affection, less frequently in the form of circumscribed tumors. It appears first in the shape of small tubercles the size of a hemp seed, either isolated or in groups; they are transparent or jelly-like. They gradually increase in size and coalesce into large irregular plagues, so that the peritoneum soon appears covered with a thick, jelly-like mass. The histological elements of the stroma vary. The coarse portion consists of distinct and strong connective tissue, which very much resembles the elastic fibrous tissue of the lungs, and when fully developed, contains but few cellular elements.

The finer portion is composed of a more uniform homogeneous or slightly fibrillated structure, and not infrequently contains round or oval nuclei and spindle shaped cells, with long projections, so that the finest network presents the appearance of being composed of arborescent and anastomosing connective tissue cells. The vascularity of the stroma varies in different cases. It has been frequently observed that during the first stages of the disease there is marked hyperæmia around the individual centers of development, which undoubtedly is due to the genesis of new blood-vessels. The capillary vessels increase in size and multiply in number by the budding process. As a general rule the soft forms of areolar cancer are more vascular than the more compact ones. At an advanced stage of the development of the growth the vessels get destroyed. The chemical composition, according to Frerichs is similar to the synovia containing albuminate of soda.

Virchow denies the protein nature of this substance, and believes that mucus is the only material found in the normal condition of the body, bearing any chemical resemblance to the colloid substance. A microscopical examination of the colloid material demonstrates that it is composed of an amorphous mass, containing different kinds of cell structures, fat globules, either free or in cells, pigment molecules and pieces of stroma.

John Mueller, Virchow and Luschka found also fat crystals and cholestrin. The cells are conspicuously large, oval, or round, and contain a hyaline, tenacious substance, one or two nuclei, and sometimes a number of endogenous cells. The diameter of the cells range from 1-100 to 1-30 of a line ; that of the nuclei from 1-200 to 1-70. In some cases the cells are similar to those found in medullary cancer. In some only the intercellular substance possesses a mucous or jelly-like property. Sometimes we find enormous cells at the periphery of the growth, appearing to the naked eye like macerated sage, containing a number of endogenous cells.

